# Hypnotic Medication

**Published:** 1887-09

**Authors:** 


### *HYPNOTIC MEDICATION.*

Hypnotic medication is addressed only to the symptoms of insomnia and excessive erethism, which, in many cases, will produce disastrous results if not relieved. These symptoms may be produced by a number of distinct diseases, and in each case a special hypnotic must be chosen, although all hypnotics produce sleep in the same manner, viz., by producing anæmia of the cerebro-spinal axis.

*Chloral* is the most powerful of hypnotics, and is indicated where the patient is of a sanguine temperament and robust constitution. On account of its irritating properties, it should not be used in gastrointestinal disorders or when the heart is diseased, except in cases of active cardiac hypertrophy.

Paraldehyde may be employed as an adjuvant of chloral, and in cases where chloral is contra-indicated. It may be employed in convulsive diseases.

Hypnome, although its hypnotic powers are doubted by some physicians, will, according to Dujardin-Beaumetz, produce sleep in cases of cephalalgia, and meets some special cases in psychiatric practice.

Urethan should be used as a hypnotic is cases of heart disease and the treatment of insomnia in children.—*Revista Med. de Sevilla.*

